# The impact of forensic experience on postmortem CT interpretation in firearm deaths: an interobserver reliability study

**DOI:** 10.1007/s11547-025-02126-4

**Published:** 2025-10-24

**Authors:** Maria Grazia Fornasari, Mauro Midiri, Giuseppe Davide Albano, Marika Triscari Barberi, Ginevra Malta, Giovanni Roccella, Roberto Cannella, Stefania Zerbo, Antonina Argo, Giuseppe Lo Re

**Affiliations:** 1 Radiology Department, Department of Biomedicine, Neuroscience and Advanced Diagnostics, University of Palermo, Palermo, Italy; 2 Department of Health Promotion, Mother and Child Care, Internal Medicine and Medical Specialties, Institute of Legal Medicine, University of Palermo, Via del Vespro 129, 90100 Palermo, Italy

**Keywords:** PMCT, Forensic radiology, Gunshot wounds, Forensic issue, Diagnostic accuracy, Inter-rater reliability

## Abstract

Forensic radiology training significantly enhances the diagnostic accuracy of postmortem computed tomography (PMCT) in firearm-related deaths, reducing inter-reader variability and improving injury detection. This study examines the impact of forensic expertise on PMCT interpretation, evaluating whether specialized training supersedes clinical radiology experience (non-forensic). A retrospective observational analysis was conducted at the University of Palermo, examining 10 firearm fatalities (homicides or suicides) between 2021 and 2024. The sample included individuals aged 25 to 66, with injuries from both short- and long-barrel firearms. Four radiologists with varying forensic experience analyzed the PMCT scans: an experienced forensic radiologist, an experienced clinical radiologist without forensic training, a radiology resident with forensic training, and a radiology resident without forensic expertise. Findings were compared against autopsy results as the gold standard. A lesion-based analysis was carried out in performance metrics considering the total number of findings (*n* = 960) and the number of findings in each subgroup (ranging from 40 up to 230 lesions). Inter-rater agreement was assessed using Fleiss’ kappa and Cohen’s kappa, while diagnostic performance was evaluated with ROC curve analysis. Results showed significantly higher diagnostic accuracy among radiologists with forensic training, particularly in detecting entrance and exit wounds, as well as organ injuries. These findings underscore the critical role of forensic radiology training in enhancing PMCT reliability, particularly for firearm-related injuries. Standardized reporting protocols and structured training programs are crucial for strengthening medicolegal investigations, thereby ensuring accurate and reproducible forensic imaging assessments. Future research should explore advanced imaging techniques, including radiomics and AI-driven analysis, to optimize forensic radiology practices.

## Introduction

Forensic radiology has become a fundamental tool in postmortem investigations, particularly in cases involving firearm injuries [1]. The introduction of postmortem computed tomography (PMCT) has transformed the field by providing a noninvasive, rapid, and highly detailed method for examining the deceased [2, 3]. This study compares the diagnostic accuracy of radiologists and residents in detecting firearm injuries via PMCT, emphasizing the impact of forensic training on diagnostic accuracy [4]. Research conducted at the Policlinico “Paolo Giaccone” in Palermo investigates whether radiologists with forensic training are more effective at identifying organ damage and bullet trajectories.

PMCT has gained significant relevance in forensic investigations due to its ability to document foreign bodies, analyze skeletal fractures, assess organ damage, and reconstruct bullet trajectories [5, 6]. It allows for the digital preservation of findings, facilitates 3D reconstructions, and proves particularly useful when cultural or religious beliefs discourage invasive procedures [7, 8]. While traditional autopsy remains the gold standard in forensic pathology, it presents some limitations, including its invasiveness, time consumption, and the challenges associated with cultural acceptance. PMCT offers compelling support by providing a faster, more widely accepted, and minimally invasive complementary approach to postmortem examinations. The technique has proven particularly effective in detecting fractures, pneumothorax, and retained foreign bodies, often identifying findings that might be overlooked in a conventional autopsy [5, 9–11].

Firearm injuries, in particular, pose a significant challenge in forensic radiology due to the complexity of wound morphology, projectile trajectories, and the varying degrees of tissue damage [12–14]. PMCT allows forensic experts to assess the location and number of wounds, determine bullet trajectories, and evaluate associated skeletal and soft-tissue damage [15–18]. Understanding firearm injuries through PMCT requires technical expertise and familiarity with forensic principles, making the integration of forensic radiology into standardized training protocols essential for the future of medicolegal investigations [19].

This pilot study highlights the importance of integrating forensic radiology into medicolegal diagnostics by analyzing various cases from the Institute of Legal Medicine of the University of Palermo. The findings aim to contribute to the standardization of PMCT methodologies, ultimately improving forensic investigations [20–22].

While PMCT has demonstrated high utility in detecting firearm-related injuries, its diagnostic reliability depends largely on the expertise of the interpreting radiologist. Previous studies have highlighted substantial interobserver variability, underscoring the influence of specialized training on diagnostic performance [23–25]. However, the extent to which forensic imaging training improves accuracy compared to conventional clinical radiology experience remains insufficiently explored.

The primary objective of this study is to assess how forensic radiology training impacts the diagnostic accuracy of PMCT in firearm-related deaths. Specifically, we compare radiologists with forensic imaging expertise to those with only clinical radiology experience, evaluating diagnostic accuracy, interobserver agreement, and lesion detection against autopsy findings as the gold standard. This study aims to contribute to the development of standardized training curricula and structured reporting protocols that enhance the reproducibility and reliability of forensic radiology assessments.

## Material and methods

This pilot study adopts a single-center, observational, retrospective design based on PMCT cases conducted at the Policlinico “Paolo Giaccone” in Palermo between January 2021 and August 2024. On average, PMCT was performed 24 h before the autopsy, ensuring optimal temporal correlation between radiological imaging and anatomical–pathological findings. For all cases, the PMI of the included cases was less than 72 h, a parameter considered suitable for ensuring adequate image quality and correct forensic interpretation.

The imaging was performed using a 128-slice MDCT scanner (Somatom Definition AS, Siemens Healthcare, Erlangen, Germany**)**, with acquisition parameters optimized for forensic applications, including thin collimation (0.6 mm, 1 mm, and 2 mm), a tube voltage of 120 kVp, and an effective tube current ranging from 120 to 160 mA. Images were anonymized and analyzed using dedicated Singovia workstations (Siemens Healthcare, Erlangen, Germany)**.** Axial and multiplanar reconstructions were reviewed using bone and lung windows, while 3D reconstructions were performed using specialized software to enhance the visualization of anatomical structures, as in previous studies [26, 27].

The t study sample consisted of 10 gunshot-related deaths occurring in Palermo and its district from 2021 to 2024. All firearm-related deaths that underwent both pre-autopsy PMCT and medicolegal autopsy at our institution between January 2021 and August 2024 were reviewed. Cases were included when a complete autopsy report was available, the PMCT had been performed before autopsy, and the postmortem interval was within 72 h to ensure adequate image quality and reliable interpretation. Exclusion criteria were advanced decomposition, incomplete imaging datasets, or missing autopsy documentation. In total, ten cases satisfied these requirements and were included in the present analysis.

The selected cases included both homicidal (eight cases) and suicidal (two cases) firearm injuries**,** with victims aged between 25 and 66 years (eight males and two females). Nine cases involved short-barreled firearms, while one involved a long-barreled firearm. Each case was evaluated through PMCT imaging and forensic autopsy reports, which served as the gold standard for lesion documentation and diagnosis. The study sought to identify injuries that might have been overlooked in the autopsy, particularly fractures in the face, vertebrae, and limbs.

### Interobserver analysis

Four radiologists independently analyzed the PMCT scans, each issuing an individual report. The group included two senior radiologists with over 20 years of experience—one specialized in forensic radiology, having performed more than 500 PMCTs, and one clinical radiologist without forensic expertise (*R*1 and *R*2, respectively). Additionally, two radiology residents participated—one with two years of forensic experience and more than 50 PMCTs performed, and one without prior forensic training (*R*3 and *R*4, respectively). All readers interpreted PMCT images independently and were blinded to autopsy findings and to the assessments of the other readers.

A structured data collection tool, using Google Sheets, was developed to ensure systematic and standardized reporting, combining a mixed-format table with anatomical body maps adapted from traditional autopsy charts. The table included a binary yes/no section to document the presence or absence of fractures, organ injuries, vascular damage, hemorrhages, entrance and exit wounds, and retained bullets in the various body districts. A free-text section allowed radiologists to describe the precise location of injuries in greater detail. Anatomical body maps were created with the support of an OpenAI-based illustration tool (https://chatgpt.com/), used exclusively to generate schematic overlays for visualization purposes, distinguishing between different types of injuries and facilitating more accurate communication between radiologists and forensic pathologists. The tool was not employed for image interpretation or for any part of the data analysis.

### Statistical analysis

This pilot study primarily aimed to explore the influence of forensic training on PMCT interpretation and generate hypotheses for future research. Categorical variables were expressed as absolute numbers and percentages, whereas continuous variables were summarized using the median and interquartile range (IQR). Interobserver agreement among the four readers was evaluated using Fleiss’ kappa, while Cohen’s kappa was calculated for pairwise comparisons between forensic-trained and clinically trained radiologists. Interpretation of agreement followed standard guidelines (poor < 0.00, slight 0.01–0.20, fair 0.21–0.40, moderate 0.41–0.60, substantial 0.61–0.80, almost perfect 0.81–1.00). Diagnostic accuracy was assessed with autopsy findings as the reference standard. Performance metrics included the area under the receiver operating characteristic curve (AUC), sensitivity, and specificity, each reported with 95% confidence intervals (CIs). Lesion-based analysis was carried out across the total sample (*n* = 960 findings) and in subgroups ranging from 40 to 230 lesions. To compare diagnostic performance, AUC values were tested using DeLong’s method, with statistical significance set at *p* < 0.05. Given the large number of pairwise AUC comparisons (>40), the risk of type I error due to multiplicity must be considered. While no formal correction was applied because of the exploratory nature of this pilot study, this limitation is explicitly discussed. All statistical computations were performed using IBM SPSS software (version 26.0, Armonk, NY, USA: IBM Corp) and MedCalc statistical software (version 14.8.1, Ostend, Belgium) to ensure rigorous data analysis.

### Ethics

This retrospective study used exclusively postmortem data. Our investigations were conducted in accordance with the principles outlined in the Declaration of Helsinki, as revised in 2013. The study was conducted in accordance with the Institutional Review Board and the Ethics Committee protocol approved by “Comitato Etico Locale Palermo 1” (N. 11—05.05.2025). Because summoning the parents was not possible, as it would badly interfere with the grieving process, the parents’ consent was completely waived according to the Italian Authority of Privacy and Data Protection (GDPR nr 679/2016; 9/2016 and recent law addition number 424/19 July 2018). Data were fully anonymized before analysis.

## Results

The frequency of lesions identified by each reader and the reference standard (autopsy) is summarized in Table 1. The study systematically evaluated 960 anatomical findings categorized by lesion type. Among these, 150 cases (15.6%) involved organ-related injuries, while 230 cases (24.0%) were associated with fractures**.** Additionally, 40 findings (4.2%) were related to vascular damage, and 50 cases (5.2%) involved hemorrhages. Firearm injuries constituted a significant portion of the dataset, with 220 cases (22.9%) of entry wounds, 190 cases (19.8%) of exit wounds, and 80 instances (8.3%) of retained bullets. This comprehensive dataset enabled a detailed comparison of reader performance across different lesion types, highlighting the impact of forensic training on diagnostic accuracy.

**Table 1:** PMCT findings by category according to the four readers, the autopsy reference standard, and reader agreement

Lesions	*R*1	*R*2	*R*3	*R*4	Autopsy	Agreement	Experience	General
All lesions	140/960 (14.6%)	82/960 (8.5%)	129/960 (13.4%)	147/960 (15.3%)	173/960 (18.0%)	0.66	0.88	0.53
Organs	25/150 (16.7%)	11/150 (7.3%)	20/150 (13.3%)	25/150 (16.7%)	29/150 (19.3%)	0.52	0.77	0.51
Fractures	46/230 (20.0%	28/230 (12.2%	45/230 (19.6%)	56/230 (24.3%)	48/230 (20.9%)	0.76	0.96	0.57
Vascular lesions	4/40 (10.0%)	3/40 (7.5%)	4/40 (10.0%)	7/40 (17.5%)	4/40 (10.0%)	0.42	0.72	0.33
Effusions	17/50 (34.0%	15/50 (30.0%)	17/50 (34.0%	17/50 (34.0%)	21/50 (42.0%)	0.80	0.91	0.73
Entry wounds	25/220 (11.4%)	11/220 (5.0%)	19/220 (8.6%)	17/220 (7.7%)	37/220 (16.8%)	0.55	0.80	0.32
Exit wounds	13/190 (6.8%)	4/190 (2.1%)	14/190 (7.4%)	13/190 (6.8%)	24/190 (12.6%)	0.36	0.88	0.21
Retained bullets	10/80 (12.5%)	10/80 (12.5%)	10/80 (12.5%)	12/80 (15.0%)	10/80 (12.5%)	0.84	1.00	0.68

The analysis of inter-reader agreement revealed that the overall concordance among the four radiologists was substantial, with a Fleiss’ kappa value of 0.66. However, the level of agreement varied significantly based on forensic experience. Radiologists with prior PMCT training demonstrated an almost perfect agreement (Cohen’s kappa = 0.88), whereas those without forensic experience showed only moderate agreement (Cohen’s kappa = 0.53).

Among the different findings, the lowest agreement was observed in identifying exit wounds, with a Fleiss’ kappa value of 0.36. Conversely, the highest agreement was recorded for retained bullets, with a Fleiss’ kappa value of 0.84. This trend suggests that more complex and subtle findings, such as exit wounds, may be more challenging to identify consistently, particularly for those without forensic radiology training. Readers with PMCT experience consistently demonstrated better agreement compared to their non-forensic-trained counterparts.

**Fig. 1:**
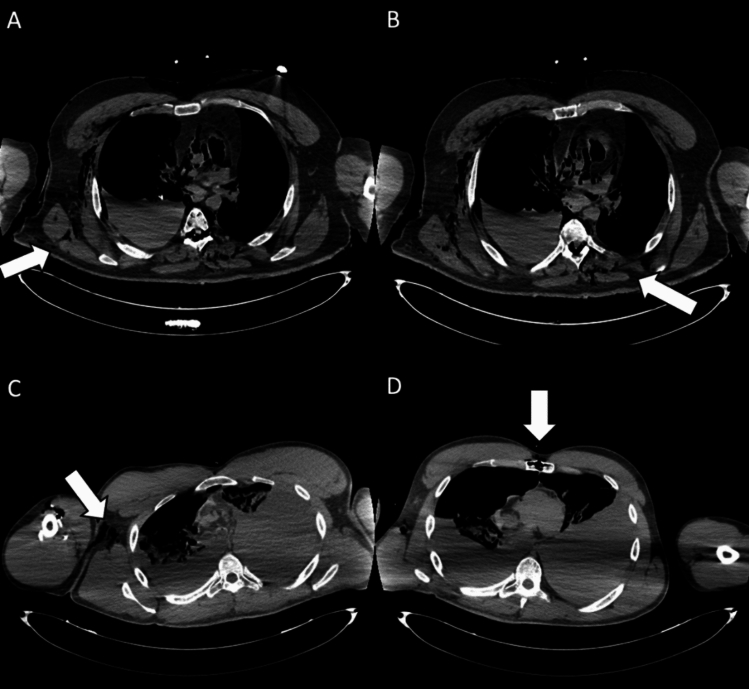
Entry wounds. **A** Axial CT scan showing an entrance wound in the right posterior thoracic wall (white arrow), with adjacent soft-tissue disruption and subcutaneous gas, consistent with a penetrating gunshot injury. **B** Axial CT image depicting the ballistic trajectory with paravertebral left soft-tissue damage (white arrow) and subcutaneous emphysema in the posterior right chest wall. **C** Cross-sectional CT slice demonstrating an entrance wound at the root of the right arm (white arrow), associated with soft-tissue laceration and subcutaneous emphysema. **D** CT image illustrating an entrance wound at the sternal level (white arrow), with anterior chest wall disruption and surrounding soft-tissue alterations, compatible with penetrating trauma. The identification of entrance gunshot wounds showed only moderate inter-reader agreement (Fleiss’ *k* = 0.55). In particular, less forensic-experienced readers demonstrated markedly reduced sensitivity (29.7%), highlighting the diagnostic challenges of detecting these findings on PMCT

The diagnostic performance based on reader experience is presented in Table 2, and it was assessed using ROC curves and AUC values (Fig. 1). The most experienced forensic radiologist (*R*1) achieved the highest overall accuracy, with an AUC of 0.898 (95% CI 0.887–0.916), followed by the radiology resident with forensic experience (*R*3), who obtained an AUC of 0.862 (95% CI 0.839–0.883). In contrast, the radiologist with clinical but no forensic training (*R*2) and the radiology resident without forensic experience (*R*4) had lower AUC values, both reaching 0.756 (95% CI 0.727–0.783).

**Table 2:** Diagnostic performance of each reader for PMCT findings

Lesions	AUC (95% CI)	*P* value	Sensitivity (95% CI)	Specificity (95% CI)
All lesions				
* R*1	0.898 (0.887, 0.916)	<0.0001	79.8 (73.0, 85.5)	99.7 (99.1, 100)
* R*2	0.716 (0.686, 0.744)	<0.0001	43.9 (36.4, 51.7)	99.2 (98.3, 99.7)
* R*3	0.862 (0.839, 0.883)	<0.0001	72.8 (65.6, 79.3)	99.6 (98.9, 99.9)
* R*4	0.756 (0.727, 0.783)	<0.0001	57.2 (49.5, 64.7)	93.9 (92.0, 95.5)
Organ injuries				
* R*1	0.910 (0.852, 0.950)	<0.0001	82.8 (64.2, 94.2)	99.2 (95.2, 100)
* R*2	0.647 (0.565, 0.723)	0.0009	31.0 (15.3, 50.8)	98.4 (94.2, 99.8)
* R*3	0.823 (0.753, 0.881)	<0.0001	65.5 (45.7, 82.1)	99.2 (95.5, 100)
* R*4	0.717 (0.638, 0.788)	<0.0001	51.7 (32.5, 70.6)	91.7 (85.3, 96.0)
Fractures				
* R*1	0.979 (0.951, 0.993)	<0.0001	95.8 (85.7, 99.5)	100 (98.0, 100)
* R*2	0.792 (0.733, 0.842)	<0.0001	58.3 (15.3, 50.8)	100 (98.0, 100)
* R*3	0.956 (0.920, 0.978)	<0.0001	91.7 (80.0, 97.7)	99.5 (97.0, 100)
* R*4	0.899 (0.853, 0.935)	<0.0001	87.5 (74.8, 95.3)	92.3 (87.4, 95.7)
Vascular lesions				
* R*1	0.861 (0.715, 0.950)	0.0041	75.0 (19.4, 99.4)	97.2 (85.5, 99.9)
* R*2	0.736 (0.573, 0.863)	0.1035	50.0 (6.8, 93.2)	97.2 (85.5, 99.9)
* R*3	1.000 (0.912, 1.000)	<0.0001	100 (39.8, 100)	100 (90.3, 100)
* R*4	0.819 (0.666, 0.923)	0.0124	75.0 (19.4, 99.4)	88.9 (73.9, 96.9)
Effusion				
* R*1	0.905 (0.788, 0.969)	<0.0001	81.0 (58.1, 100)	100 (88.1, 100)
* R*2	0.775 (0.635, 0.881)	<0.0001	61.9 (38.4, 81.9)	93.1 (77.2, 99.2)
* R*3	0.864 (0.737, 0.944)	<0.0001	76.2 (58.2, 91.8)	96.6 (82.2, 99.9)
* R*4	0.864 (0.737, 0.944)	<0.0001	76.2 (52.8, 91.8)	96.6 (82.2, 99.9)
Entry wounds				
* R*1	0.838 (0.782, 0.884)	<0.0001	67.6 (50.2, 82.0)	100 (98.0, 100)
* R*2	0.649 (0.582, 0.712)	0.0001	29.7 (15.9, 47.0)	100 (98.0, 100)
* R*3	0.757 (0.695, 0.812)	<0.0001	51.4 (34.4, 68.1)	100 (98.0, 100)
* R*4	0.632 (0.565, 0.696)	0.0006	29.7 (15.9, 47.0)	93.7 (93.0, 98.8)
Exit wounds				
* R*1	0.771 (0.704, 0.829)	<0.0001	54.2 (32.8, 74.4)	100 (97.8, 100)
* R*2	0.559 (0.486, 0.631)	0.0857	12.5 (2.7, 32.4)	99.4 (96.7, 100)
* R*3	0.792 (0.727, 0.847)	<0.0001	58.3 (36.6, 77.9)	100 (97.8, 100)
* R*4	0.632 (0.565, 0.696)	0.0006	16.7 (4.7, 14.2)	100 (97.8, 100)
Retained bullets				
* R*1	1.000 (0.995, 1.000)	<0.0001	100 (69.2, 100)	100 (94.9, 100)
* R*2	1.000 (0.995, 1.000)	<0.0001	100 (69.2, 100)	100 (94.9, 100)
* R*3	1.000 (0.995, 1.000)	<0.0001	100 (69.2, 100)	100 (94.9, 100)
* R*4	0.871 (0.778, 0.936)	<0.0001	80.0 (44.4, 97.5)	94.3 (86.0, 100)

**Fig. 2:**
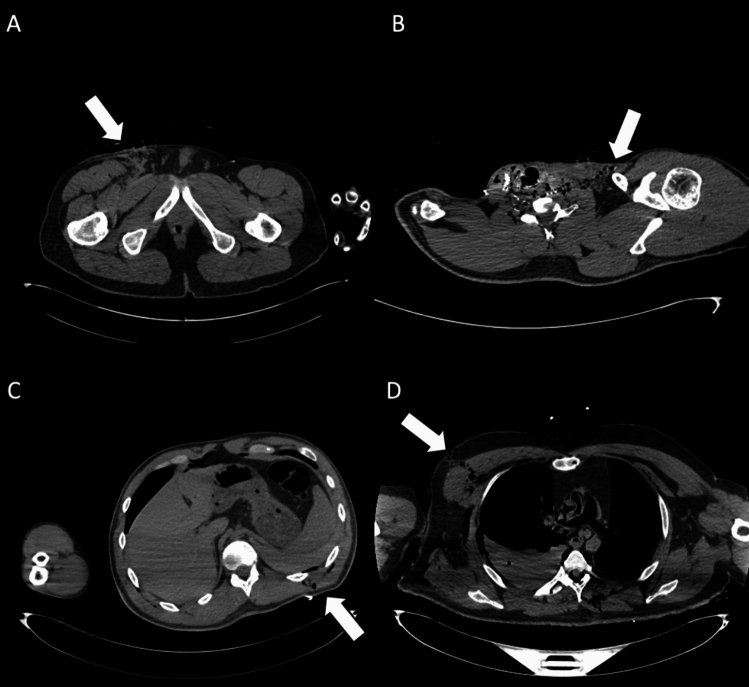
Exit wounds. **A** Axial CT scan showing an exit wound in the subcutaneous soft tissues at the root of the right thigh (white arrow). **B** Axial CT image depicting an exit wound in the left supraclavicular region (white arrow), visible as a focal soft-tissue defect with surrounding gas bubbles. **C** Cross-sectional CT slice illustrating an exit wound at the left posterior abdominal wall (white arrow), with subcutaneous emphysema and adjacent soft-tissue disruption. **D** CT image demonstrating an exit wound in the right chest region (white arrow), characterized by soft-tissue laceration and subcutaneous gas. Exit gunshot wounds were associated with the lowest inter-reader agreement (Fleiss’ *k* = 0.36). Despite the very high specificity of virtopsy, sensitivity was particularly low among readers without forensic experience (12.5% and 16.7%), underlining the difficulty in correctly identifying these injuries on PMCT

**Fig. 3:**
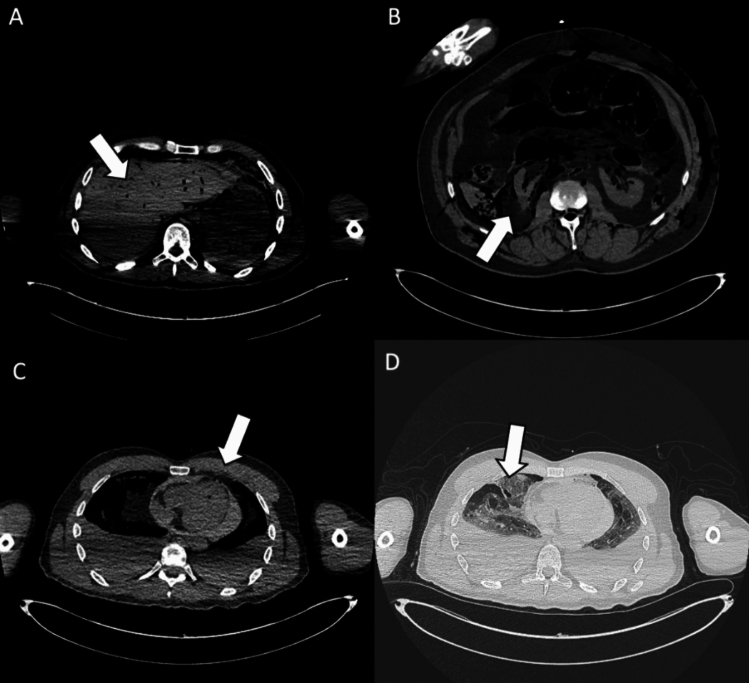
Organ injuries. **A** Axial CT scan showing traumatic injury to the bi-lobar liver parenchyma (white arrow). Moderate postmortem decomposition is also evident, with heterogeneous intraparenchymal gas distribution. **B** Cross-sectional CT image demonstrating a laceration of the right kidney (white arrow), consistent with penetrating trauma. **C** Axial CT slice showing a pericardial lesion (white arrow), with small gas bubbles in the anterior pericardial region, indicative of traumatic pericardial injury. **D** CT image highlighting a laceration of the right middle lobe (white arrow), associated with pulmonary contusion, omolateral pneumothorax, and bilateral pleural effusion. In the assessment of organ injuries, inter-reader agreement was only moderate (Fleiss’ *k* = 0.52). Sensitivity was again limited among less forensic-experienced readers (31.0% and 51.7%), reflecting the interpretative complexity and reduced diagnostic accuracy of PMCT in this setting

Although the specificity of PMCT evaluation remained consistently high across all readers (≥99.2%), sensitivity varied significantly depending on the reader’s experience. Readers without forensic expertise (*R*2 and *R*4) showed low sensitivity in detecting key findings. For organ injuries, sensitivity was only 31.0% for *R*2 and 51.7% for *R*4. Similarly, for firearm entry wounds, both *R*2 and *R*4 had a sensitivity of 29.7%, while for exit wounds, their sensitivity was even lower, at 12.5% (*R*2) and 16.7% (*R*4). These results suggest that forensic training plays a crucial role in improving the detection of complex findings such as internal injuries and gunshot wounds (Figs. 1, 2, 3).

A comparison of diagnostic performance across different experience levels revealed statistically significant differences (Table 2). However, no significant differences were observed between the two readers with forensic training (*R*1 and *R*3) in detecting fractures (*p* = 0.1118), vascular injuries (*p* = 0.2695), hemorrhages (*p* = 0.1626), exit wounds (*p* = 0.5692), or retained bullets (*p* = 1.000). This suggests that even limited forensic training, such as that of a radiology resident with PMCT experience, can significantly improve diagnostic accuracy, bringing performance levels closer to those of an experienced forensic radiologist.

These findings underscore the crucial role of forensic training in enhancing the accuracy of PMCT.

**Fig. 4:**
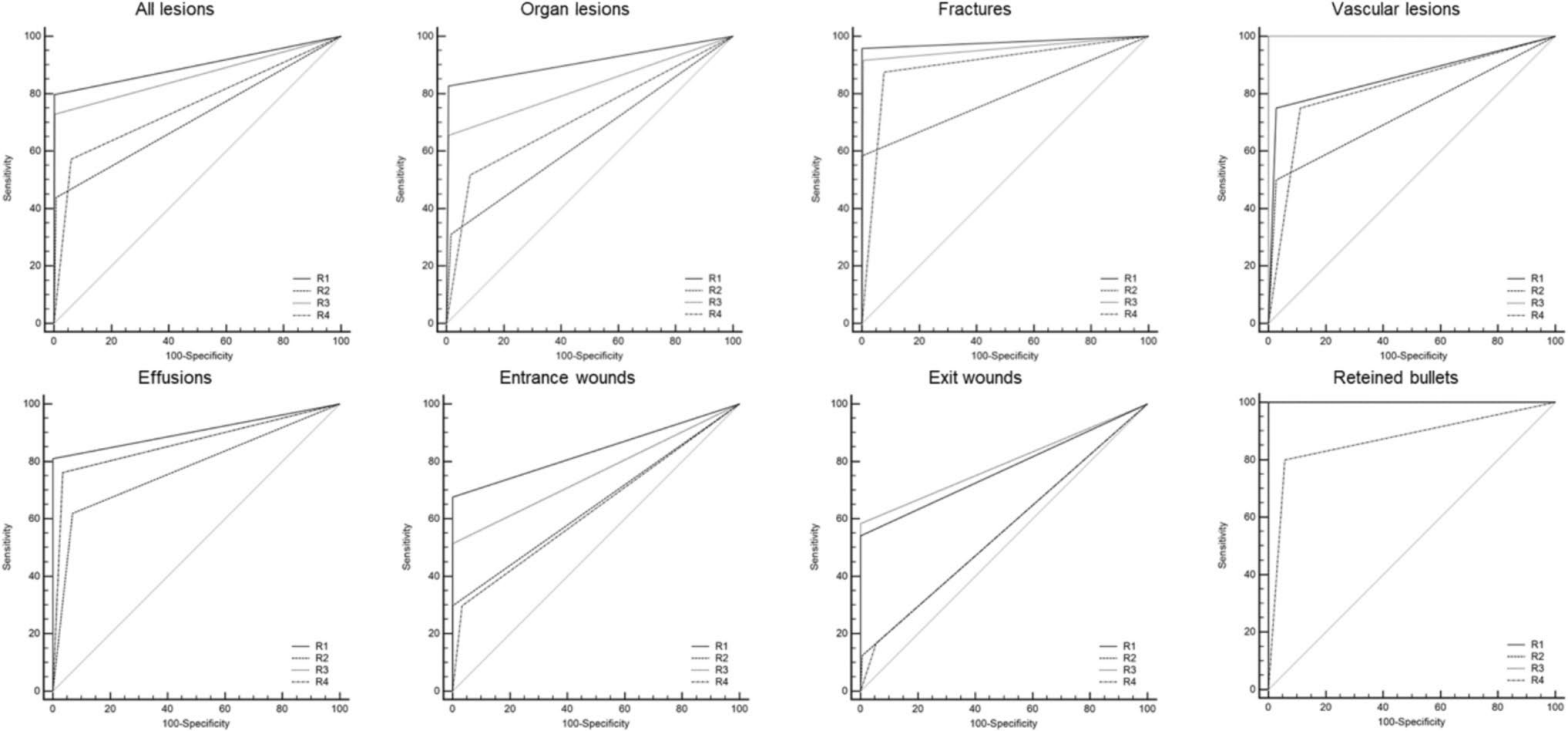
ROC curve (receiver operating characteristic) based on the reader’s experience

The less experienced readers (*R*2 and *R*4) struggled to identify injuries, particularly entry and exit wounds and internal organ damage. Radiologists with forensic expertise (*R*1, *R*3) consistently outperformed their non-expert counterparts, demonstrating that forensic radiology training significantly improves postmortem imaging diagnostic performance (Fig. 4).

## Discussion

This pilot study demonstrated that forensic radiology training improves diagnostic performance, even in radiologists with limited clinical experience [28]. The analysis of 960 findings revealed significant differences in accuracy between radiologists trained in PMCT and those without specific forensic expertise. The overall inter-reader agreement was classified as substantial (Fleiss’ kappa = 0.66), with nearly perfect agreement among readers with forensic experience (Cohen’s kappa = 0.88) and moderate agreement among those without such training (Cohen’s kappa = 0.53). The highest agreement was observed in detecting retained bullets (Fleiss’ kappa = 0.84), while the lowest was recorded for identifying exit wounds (Fleiss’ kappa = 0.36) (Table 3).

**Table 3:** Comparison of diagnostic performance based on reader experience

Lesions	*R*1 versus *R*2	*R*1 versus *R*3	*R*1 versus *R*4	*R*2 versus* R*3	*R*2 versus* R*4	*R*3 versus* R*4
All lesions	<0.0001	0.0085	<0.0001	<0.0001	0.0390	<0.0001
Organ injuries	<0.0001	0.0494	0.0002	0.0007	0.0811	0.0327
Fractures	<0.0001	0.1118	0.0004	<0.0001	0.0044	0.0136
Vascular lesions	0.6028	0.2695	0.8400	0.0688	0.7290	0.1577
Hemorrhage	0.0095	0.1626	0.3651	0.0381	0.0729	1.0000
Entry wounds	<0.0001	0.0256	<0.0001	0.0016	0.7322	0.0050
Exit wounds	<0.0001	0.5692	0.003	<0.0001	0.9312	0.0001
Retained bullets	1.0000	1.0000	0.0591	1.0000	0.0591	0.0591

The table presents the *p* values obtained using DeLong’s test, which evaluates the statistical significance of differences in diagnostic performance between pairs of readers

The diagnostic performance, assessed through the area under the curve (AUC), confirmed that radiologists with forensic training (*R*1 and *R*3) outperformed those with only clinical experience but no forensic training. Their respective AUC values (0.898 and 0.862) were significantly higher than those of radiologists without forensic expertise (*R*2: 0.716; *R*4: 0.756). These findings reinforce the importance of PMCT training in enhancing diagnostic skills, particularly in detecting complex injuries such as organ damage, entry wounds, and exit wounds [16, 29].

The results of this study underscore the significant impact of forensic radiology training on the accuracy and reliability of postmortem imaging interpretation [30, 31]. The most robust results are the interobserver agreement (kappa values), supported by descriptive statistics of lesion detection. The superior performance of radiologists with forensic training suggests that specific expertise in PMCT enhances the ability to identify injuries with greater precision [32, 33]. In particular, firearm-related injuries, such as entry and exit wounds, showed a significant difference in detection accuracy between radiologists with forensic training and those without. This finding is consistent with previous studies, highlighting how experience in forensic radiology significantly reduces inter-reader variability and improves the identification of subtle traumatic findings [29]. For example, previous studies have emphasized that radiologists with forensic training have greater diagnostic confidence when assessing ballistic trauma, particularly in differentiating entry from exit wounds [34]. Other studies have similarly reported that forensic expertise is essential in interpreting gunshot-related injuries in postmortem CT scans [35–39]. The lower accuracy of radiologists without forensic expertise in identifying entry and exit wounds aligns with previous forensic imaging studies. Research suggests that interpreting wound trajectories and subtle fractures requires specialized training. The high agreement among radiologists with forensic training supports the idea that expertise leads to more standardized and reliable diagnoses, reinforcing the need to integrate forensic radiology training into radiology curricula [40].

Despite its insights, this study has limitations. A larger dataset, including cases with broader postmortem intervals, would provide a better understanding of the effects of decomposition on imaging interpretation. The case sample was small (*n* = 10), from a single center over a limited time frame, and may not reflect the full heterogeneity of firearm-related deaths in terms of weapon type, injury spectrum, or postmortem interval. Furthermore, these were judicial autopsies, and therefore the sample size was not predictable, as it strictly depended on the number of firearm-related crimes occurring during the study period. The opportunistic nature of case selection further limits representativeness. Given the large number of pairwise AUC comparisons (>40), the risk of type I error due to multiplicity must be considered. We recognize that lesion-level analysis treats each finding as independent, whereas findings are clustered within cases and readers. This approach inflates the nominal sample size and may overestimate statistical significance. Future studies with larger case numbers should employ cluster-aware models. Future research should also explore the integration of radiomics and machine learning to enhance forensic diagnostics and reduce inter-reader variability through objective, quantitative assessments [41, 42].

Another crucial aspect is the standardization of forensic radiology training. Given its demonstrated benefits, structured training programs and certification would ensure radiologists develop the necessary skills for accurate postmortem imaging [43]. International collaborations could establish consensus guidelines**,** improving the reliability of PMCT in forensic investigations [44–46]. Furthermore, the selected study sample limits the generalizability of the findings. All the examined subjects had died within 72 h and showed relatively limited putrefactive changes. This sample type may not adequately reflect evaluators’ challenges when dealing with longer postmortem intervals or specific pathological conditions [47]. More advanced decomposition could interpret the process as more complex, potentially impacting the reliability of the issued reports [48, 49]. Larger, multicenter cohorts will be necessary to confirm these findings, increase generalizability, and explore broader forensic scenarios. Larger multicenter studies could allow for validating these observations, expanding the spectrum of firearm-related injuries investigated, and assessing the impact of different postmortem intervals and varying degrees of decomposition on PMCT interpretation. Such research would allow a more comprehensive understanding of the reliability and reproducibility of postmortem CT in forensic practice.

The major strength of this study is that forensic experience significantly improves postmortem imaging accuracy, particularly for firearm-related injuries [50]. A more extensive and varied sample would allow a more comprehensive evaluation of postmortem CT reliability in forensic casework [51–54]. Expanding forensic radiology training and integrating AI-driven analysis could further enhance accuracy and standardization in forensic investigations. Future work should also explore the integration of advanced imaging techniques, including radiomics and artificial intelligence [55, 56], which may improve diagnostic accuracy, standardize interpretations, and further reduce interobserver variability [57–61]. Furthermore, structured forensic radiology training programs should be developed to ensure that radiologists acquire the necessary expertise to interpret postmortem findings accurately [62].

Forensic radiology training significantly improves the accuracy and reliability of postmortem imaging interpretations [63]. Implementing standardized training programs and advancing forensic imaging methodologies will be crucial in optimizing the application of PMCT in forensic investigations [51, 64–66]. By fostering interdisciplinary collaboration and investing in technological advancements, forensic radiology can continue to evolve as a crucial tool in modern forensic medicine [54, 67]. Forensic radiology training not only improves diagnostic accuracy but also equips radiologists with the ability to recognize injury patterns specific to firearm-related fatalities, differentiate true lesions from postmortem artifacts, and apply medicolegal standards in structured reporting [2, 68]. Beyond individual expertise, recent reviews stress the importance of interdisciplinary collaboration, standardized curricula, and the integration of emerging technologies such as artificial intelligence and virtual autopsy to further reduce subjectivity and variability in forensic image interpretation. Advanced training frameworks that combine technological innovation with ethical and legal awareness are crucial for ensuring both the scientific validity and judicial reliability of forensic imaging findings [68]

## Conclusion

This pilot study highlights the fundamental role of forensic radiology training in improving the accuracy and consistency of postmortem imaging assessments [69, 70]. The results demonstrate that radiologists with forensic expertise are significantly more accurate in identifying firearm-related injuries, particularly entry and exit wounds, than those without specific training. The high inter-reader agreement among radiologists with forensic training reinforces the importance of postmortem imaging education, as it contributes to more standardized and reliable interpretations. These findings support the growing recognition of postmortem CT as a valuable tool in forensic investigations, providing a noninvasive and reproducible method for assessing injuries to support forensic expert evaluation.

## Data Availability

The data presented in this study is available on request from the corresponding author.
